# Role of Curing Temperature of Poly(Glycerol Sebacate) Substrates on Protein-Cell Interaction and Early Cell Adhesion

**DOI:** 10.3390/polym13030382

**Published:** 2021-01-26

**Authors:** Rubén Martín-Cabezuelo, José Carlos Rodríguez-Hernández, Guillermo Vilariño-Feltrer, Ana Vallés-Lluch

**Affiliations:** 1 Centre for Biomaterials and Tissue Engineering, Universitat Politècnica de València, 46022 Valencia, Spain; rubmarca@doctor.upv.es (R.M.-C.); jorodhe1@upvnet.upv.es (J.C.R.-H.); guivifel@upv.es (G.V.-F.); 2 Biomedical Research Networking Centre in Bioengineering, Biomaterials and Nanomedicine (CIBER-BBN), 46022 Valencia, Spain

**Keywords:** poly(glycerol sebacate), focal adhesion, protein adsorption, polymer-protein interaction

## Abstract

A novel procedure to obtain smooth, continuous polymeric surfaces from poly(glycerol sebacate) (PGS) has been developed with the spin-coating technique. This method proves useful for separating the effect of the chemistry and morphology of the networks (that can be obtained by varying the synthesis parameters) on cell-protein-substrate interactions from that of structural variables. Solutions of the PGS pre-polymer can be spin-coated, to then be cured. Curing under variable temperatures has been shown to lead to PGS networks with different chemical properties and topographies, conditioning their use as a biomaterial. Particularly, higher synthesis temperatures yield denser networks with fewer polar terminal groups available on the surface. Material-protein interactions were characterised by using extracellular matrix proteins such as fibronectin (Fn) and collagen type I (Col I), to unveil the biological interface profile of PGS substrates. To that end, atomic force microscopy (AFM) images and quantification of protein adsorbed in single, sequential and competitive protein incubations were used. Results reveal that Fn is adsorbed in the form of clusters, while Col I forms a characteristic fibrillar network. Fn has an inhibitory effect when incubated prior to Col I. Human umbilical endothelial cells (HUVECs) were also cultured on PGS surfaces to reveal the effect of synthesis temperature on cell behaviour. To this effect, early focal adhesions (FAs) were analysed using immunofluorescence techniques. In light of the results, 130 °C seems to be the optimal curing temperature since a preliminary treatment with Col I or a Fn:Col I solution facilitates the formation of early focal adhesions and growth of HUVECs.

## 1. Introduction

Biodegradable elastomers are becoming fundamental in the field of materials for medical applications [1,2,3]. Particularly, poly(glycerol sebacate), PGS, which is an elastomeric hyperbranched polyester proposed firstly by Wang et al. as a potential biomaterial in 2002, is nowadays one of the most studied polyesters [4,5,6]. This is because PGS possesses good biocompatibility and biodegradability, in addition to its versatile chemical and mechanical properties [7,8,9,10]. All of this enables PGS usage as a scaffolding material in biomedical and tissue engineering applications, such as drug delivery [11,12] and tissue regeneration of cartilage, nerve, cardiac muscle and bone [13,14,15,16,17,18,19,20], among others.

Some of the most common techniques for understanding polymer-tissue characterisation and interaction are chemical and biological polymer functionalisation [13,14]. Proteins such as fibronectin (Fn), collagen, laminin and vitronectin, all of them components of the extracellular matrix (ECM), are commonly used for biological characterisation of biomaterials [15]. Each tissue has a specific ECM composition tailored to its physiological needs. This specificity is achieved thanks to the cross-talking between several biochemical and biophysical cellular constituents, and its microenvironment. Some of the ECM proteins functions are to provide an adequate attachment structure for tissue morphogenesis, differentiation and homeostasis [16]. Particularly, the collagen family comprises 28 different species and has structural functions contributing to cell mechanical stimuli. Collagen type I (Col I) happens to be the prime representative of its family due to its large presence across several body tissues, specifically, on the endothelial wall of the blood vessels [17,18]. The typical Col I structure has the form of fibrils with a diameter of around 50 to 200 nm. Many three-stranded Col I molecules are needed to build the Col I fibrillar structure. There is a space of 67 nm between their adjacent fibrils, which define a characteristic Col I pattern of bands [19]. On the other hand, Fibronectin (Fn) is a glycoprotein present in blood, connective tissues and extracellular fluids and is a component of cell surfaces. Fn structure is formed by two subunits of 220 kDa that play a central role in cell adhesion [20,21].

These two proteins, Col I and Fn, are directly related to the adhesive cell interactions with their substrates and local microenvironments, which are fundamental for the understanding of biomaterial-cells interactions [18,22,23,24]. The main type of cell-adhesion receptor protein, called integrin, binds to extracellular matrix ligands, thus providing anchor points for the cytoskeleton organisation of the cells, called focal adhesion (FA). Integrins play an important role not only in early cell adhesion via the formation of FAs, but regarding cell migration and its fate, acting as a major fibronectin receptor on most cells. Hence, FAs are usually studied for biomaterial characterisation [22,25,26].

Moreover, it has been proved that Col I and Fn incubated together on polymer surfaces generate a synergistic effect leading to better support for endothelialisation [27]. The understanding of cell-protein-material signalling behaviour is one of the goals of this study. This could be achieved thanks to the development of a PGS coating procedure, which facilitates studying the PGS-protein-cell surface interaction without the interference of any other surface factor.

To accomplish that, a novel synthesis procedure involving non-cured PGS spin-coating was developed. Three curing temperatures (130 °C, 150 °C and 170 °C) were applied to achieve smooth, thin films that provide no external surface influence and facilitate PGS-substrate interaction with proteins and cells. By avoiding defective, heterogenous surfaces, it was possible to characterise the physicochemical properties of the PGS substrates surfaces, with or without combinations of Col I and Fn, as major representative proteins of the ECM. For further PGS surface determination, biocompatibility and protein-cell interaction studies were performed by culturing human umbilical vein endothelial cells (HUVECs). These cells were of particular interest for their growth in an adherent monolayer. Immunofluorescence techniques highlighted the effect of different PGS curing temperatures on the early focal adhesions (FAs) of HUVECs.

## 2. Materials and Methods

### 2.1. Materials

The reagents glycerol (Gly), sebacic acid (SA), ethyl alcohol (EtOH, 99%), diethylene glycol, diiodomethane, formamide, fibronectin from human plasma (Fn), phosphate buffered saline (PBS), bovine serum albumin (BSA), triton X-100 and monoclonal antivinculin clone hVIN-1 were purchased from Merk, Madrid, Spain. All reagents were used without further purification. The rest of the biological reagents, Micro BCA™ Protein Assay Kit, secondary antibody Alexa Fluor 555 Goat anti-Mouse IgG, Alexa Fluor™ 488 Phalloidin and AlamarBlue^TM^ viability assays were purchased from Thermo Scientific™, Madrid, Spain, except 4′,6-diamidino-2-phenylindole dihydrochloride (DAPI) from Vector Laboratories and PureCol^®^ Type I Collagen Solution, 3 mg/mL (Col I) from Advanced BioMatrix. Troisdorf, Germany. Endothelial cells (HUVECs) were purchased from Cellworks, Buckingham, United Kingdom, and supplemented endothelial cell medium was provided by Innoprot, Bizkaia, Spain.

### 2.2. Synthesis of PGS Substrates

An equimolar amount of sebacic acid and glycerol (1:1) was placed in a reactor under a constant flow of N_2_ atmosphere, which allows water and evaporated glycerol to recirculate avoiding glycerol loss during its polycondensation reaction. Thus, the pre-polymerisation reaction took place at 130 °C for 24 h under constant stirring to get the PGS pre-polymer (pPGS). Subsequently, to obtain thin PGS films just a few nanometers thick, a spin-coating device (WS-650MZ-23, Laurell Technologies, North Wales, Pennsylvania, US) was used. A broad range of solution concentrations from 0.01% to 1% *w*/*v* of pPGS in EtOH were poured onto 12 mm-diameter glass coverslips and then accelerated for 30 s at 3000–25,000 rpm with an angular acceleration of 1000–3000 rpm^2^ under N_2_ in the spin-coater equipment. For the complete reaction of pPGS pastes into fully cured PGS polymeric films, three curing temperatures were then applied for 48 h in an oven: 130 °C, 150 °C and 170 °C. After cooling down, the three sets of PGS substrates (henceforth termed PGS x °C, where x stands for the curing temperature) were washed three times in ethanol/water solutions (5% *v/v*) to eliminate unreacted monomers and impurities and then dried overnight under vacuum conditions at room temperature.

### 2.3. Characterisation

Fourier transform infrared spectroscopy (FTIR) spectra of PGS substrates cured at different temperatures were collected using a Platinum ATR from an ALPHA II spectrometer (Bruker, Madrid, Spain). To obtain the respective spectra, the parameters used were 24 scans at 4 cm^−1^ resolution, between 600 and 4000 cm^−1^. The wettability of PGS substrates once cured was determined using water contact angle (WCA) measurements. Data were collected in the sessile drop mode by an OCA25 Dataphysics Instruments GmbH, Mataró, Spain, from 3 µl ultra-pure MilliQ water drops and at least ten measurements per type of elastomer. PGS substrates’ surface energy (*σ_Total_*) was calculated following the Owens, Wendt, Rabel and Kaelble model (OWRK) [28] and Young equations. In this model, two surface energy components, polar (*σ*_p_) and dispersive (*σ*_d_), determine the intermolecular surface interactions. To obtain them, five solvents (diethylenglycol, ethanol, diiodomethane, glycerol and formamide) were used along with the previous values of water angles (WCA). An atomic force microscope (AFM) (Bruker Multimode 8 model, Madrid, Spain) operating in tapping mode enabled the topography and composition of PGS substrates to be evaluated. A Bruker RFESPA model silicon tip was set up with a resonant frequency of 75 kHz, a constant spring of 3 N/m and a linear speed set at 2 µm/s. The oscillation-free length of 700 mV was applied by modifying the drive amplitude. A ratio between the amplitude set-point and the free amplitude of 0.85 was kept. Images were taken for PGS substrates and non-treated glass coverslips (as controls), with and without protein coatings. For AFM image analyses, NanoScope Analysis v1.50 (Bruker, Madrid, Spain) software was used.

### 2.4. Determination of Protein Adsorption

Series of PGS samples cured at different temperatures were sterilised with UV light for 15 min on each side prior to adsorption. Solutions of Fn and/or Col I in PBS were adsorbed on the PGS substrates. Then, 100 µL of protein solution was added for an incubation period of 10 min onto each substrate. Next, the supernatant was collected for further analysis. After protein incubation, the disks were washed twice with PBS and then dried under argon gas flow. Three protein concentrations were considered: 400 µg/mL, 80 µg/mL and 40 µg/mL for Col I and 100 µg/mL, 20 µg/mL and 10 µg/mL for Fn. Protein concentrations were selected based on previous polymer coating studies [29,30,31,32]. To analyse the effect of protein combinations on the substrates, alternance and competition assays were performed. For the alternating protein adsorption study, Fn and Col I were incubated in both possible sequences: Fn 100 µg/mL first, followed by Col I 80 µg/mL (Fn/Col I) and vice versa, Col I 400 µg/mL first, followed by Fn 20 µg/mL (Col I/Fn). These protein concentrations were chosen to ensure that a saturated protein layer could be obtained. Additionally, protein solutions were mixed at different volumetric proportions (Fn 10 µg/mL:Col I 40 µg/mL, 75:25, 50:50 and 25:75) for protein competition analyses and then incubated onto the PGS substrates. The amount of total protein adsorbed was quantified by means of the Micro BCA™ Protein Assay Kit, with BSA as standard. Glass coverslips were used as a control. Four substrate replicas of each protein condition were used. AFM was used for evaluating the topography and composition of adsorbed proteins as explained before.

### 2.5. Biological Characterisation 

HUVECs were cultured in supplemented endothelial cell medium as recommended by its supplier and maintained in an incubator at 37 °C and 5% of CO_2_. Cell culture medium was replaced every 2 days until reaching 80% of confluence when they were split. Cell passage below 10 was used for all experiments. HUVECs were plated at a density of 4000 cells·cm^−2^ on both coated and non-coated PGS substrates, cured at different temperatures, with glass coverslip as controls. For cell adhesion experiments, HUVECs were placed for 3 h in endothelial cell medium in the absence of serum and then fixed in 4% paraformaldehyde (Panreac, Barcelona, Spain) for 20 min at room temperature (RT). After fixation, samples were rinsed with PBS and stored at 4 °C. Fixed HUVECs were first permeabilised with DPBS and 0.5% Triton X-100 at RT for 5 min. Samples were blocked using DPBS and 2% BSA for 2 h at RT and then incubated with monoclonal antivinculin, clone hVIN-1 (1:400) for 2 h at RT. After three rinses in DPBS and 0.1% Triton X-100, samples were incubated for 1 h at RT with secondary antibody Alexa Fluor 555 Goat anti-Mouse IgG (1:700) and Alexa Fluor™ 488 Phalloidin (1:100). Finally, after washing twice with DPBS and 0.1% Triton X-100, samples were mounted on a glass slide with Vectashield and DAPI, and fluorescence images were taken in a Nikon Eclipse 80i microscope (Nikon Instruments Europe B.V., Barcelona, Spain). For cell viability experiments, AlamarBlue^TM^ viability assays were performed after 1, 3, 5 and 7 days of HUVECs culture on the non-coated PGS substrates and glass cover slides, to verify their adequate biocompatibility. Four replicates per sample type were used. Shortly after preparation, 20 µL of endothelial cell medium were withdrawn from each sample well and then replaced with AlamarBlue reagent and incubated for 3 h at 37 °C. Supernatants were placed in a 96-well plate to read their absorbance signal at wavelengths of 570 and 600 nm. Cell death control was needed, and for that purpose, a 10% of dimethyl sulfoxide (DMSO, Sigma Aldrich) was added to four glass cover slides cultured as previously described. Acellular glass cover slides were used as blank reagent solution for the subtraction of background absorbance.

### 2.6. Data Analysis 

Image analysis for HUVEC’s focal adhesions (FAs) was performed using the software ImageJ (National Institutes of Health, Bethesda, MD, USA) Trainable Weka Segmentation plugin to obtain a binary mask. Using the same software, FA size and cell surface area from cytoskeleton images were quantified and analysed. A minimum of ten pictures were analysed, with at least one cell per image, and means and standard deviations were calculated. GraphPad Prism8 software was used for statistical analysis. Either an unpaired two-tailed t-test or a one-way ANOVA with Tukey post-test were performed where appropriate. 

## 3. Results and Discussion

### 3.1. Effect of The Curing Temperature on The Chemical and Surface Properties of PGS Substrates

**Figure 1:**
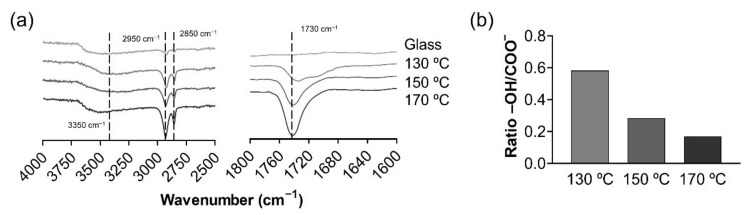
Chemical properties of poly(glycerol sebacate) (PGS) substrates. Fourier transform infrared spectroscopy (FTIR) spectra (transmittance) of PGS substrates obtained at different cured temperatures (130 °C, 150 °C and 170 °C) and glass cover slides. (**a**) Detailed FTIR spectra at the PGS characteristic chemical peak wavelengths: 1600–1800 cm^-1^ for COO^−^ peak on the right and 4000–2500 cm^−1^ for free –OH groups and -CH_2_ bonding on the left. (**b**) –OH/COO^−^ ratio obtained from transmittances at 3350 cm^−1^ and 2927 cm^−1^.

FTIR spectroscopy enabled the presence of PGS on the circular glass coverslips obtained via spin-coating and the full evaporation of the solvent to be confirmed. The FTIR spectra for PGS cured at increasing temperatures as well as for glass coverslips used as a control are represented in Figure 1. The spectra show a broad band around 3460 cm^−1^ from hydroxyl groups (O-H elongation) together with the aliphatic backbone of C–H absorption, which is located at 2950–2850 cm^−1^ (Figure 1a left). The characteristic peak of the ester carbonyl (–COO^−^) resulting from the polycondensation reaction between hydroxyl groups and free carboxyl groups of glycerol and sebacic acid, respectively, is located at 1740 cm^−1^ (Figure 1a right). None of the previous characteristic peaks are present in the spectrum of neat glass, as expected. Figure 1b presents the ratio between free hydroxyl groups and reacted carbonyl ester groups, showing a downward trend when a higher curing temperature was applied. 

All these results combined confirm the presence of PGS cured under the three curing temperatures (130 °C, 150 °C and 170 °C) using the spin-coating technique. When higher curing temperatures are applied, the reactivity of PGS oligomers and macromers increases, yielding a more crosslinked elastomer, as exposed in Figure 1b. These results are in accordance with literature for non-spin-coated PGS films [33,34].

There is a general trend regarding coating processes where hydrophobic surfaces adsorb a major amount of proteins [24]. To confirm this surface behaviour, the characterisation of PGS substrates was necessary. Therefore, Table 1 compiles the water contact angle (WCA) and surface tensions (*σ_total_*) of the three sets of substrates, the latter split into their two components, polar (*σ_p_*) and dispersive (*σ_d_*), together with their surface polarity (*σ_p_/σ_total_*). WCA values of PGS substrates are higher than the ones from glass coverslips, as expected from a relatively hydrophobic polymer [33]. The polar components of the polymeric films are significantly lower than those of the polymer-free coverslip. It can also be observed that the surface tensions of PGS 150 °C and 170 °C are slightly higher than that of PGS 130 °C, due to an increase in the dispersive component and a minor increase in the polar component in the case of PGS 170 °C, while the polar component of PGS 150 °C decreases. As for the differences in WCA values, they are only relevant (with a *p*-value below 0.0001) between glass and the PGS substrates.

### 3.2. Biological Behaviour of PGS Substrates

#### 3.2.1. Effect of PGS on HUVECs Viability and FA

**Figure 2:**
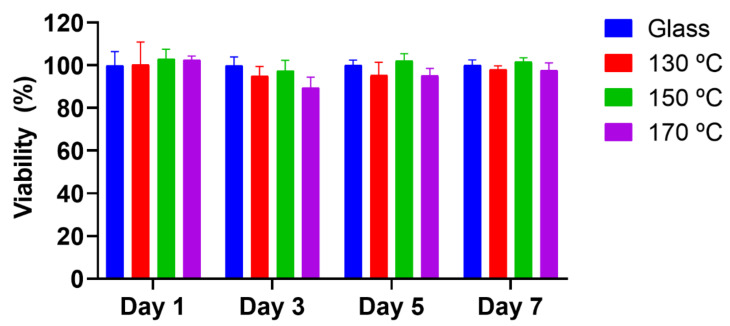
Human umbilical endothelial cells (HUVECs) biocompatibility on PGS substrates. HUVECs viability results from AlamarBlue^TM^ colorimetry technique. Glass was used as a control of good cell viability, and a 10% DMSO solution was used as cytotoxic control. Sample data distributions were analysed through a two-way ANOVA and Bonferroni post hoc multiple mean comparison test with no significant differences between samples.

Before investigating the role of PGS surfaces on protein adsorption and HUVECs early adhesion and FA formation, viability experiments on PGS substrates were performed, testing curing at different temperatures. For that, HUVECs were cultured for 7 days onto the polymer substrates and then analysed by means of AlamarBlue^TM^ colorimetry assays (Figure 2). PGS samples presented good biocompatibility, as occurred with glass coverslips. Indeed, PGS has been reported in the literature to be biocompatible [4,8], although spin-coated samples are being used in this case, which could trigger a disadvantageous performance. Due to this manufacturing process, the coatings obtained may not match in their morphology (crosslinks) or physical state (less crystallinity, for example) with their analogous films (cured at the same temperature) [33].

**Figure 3:**
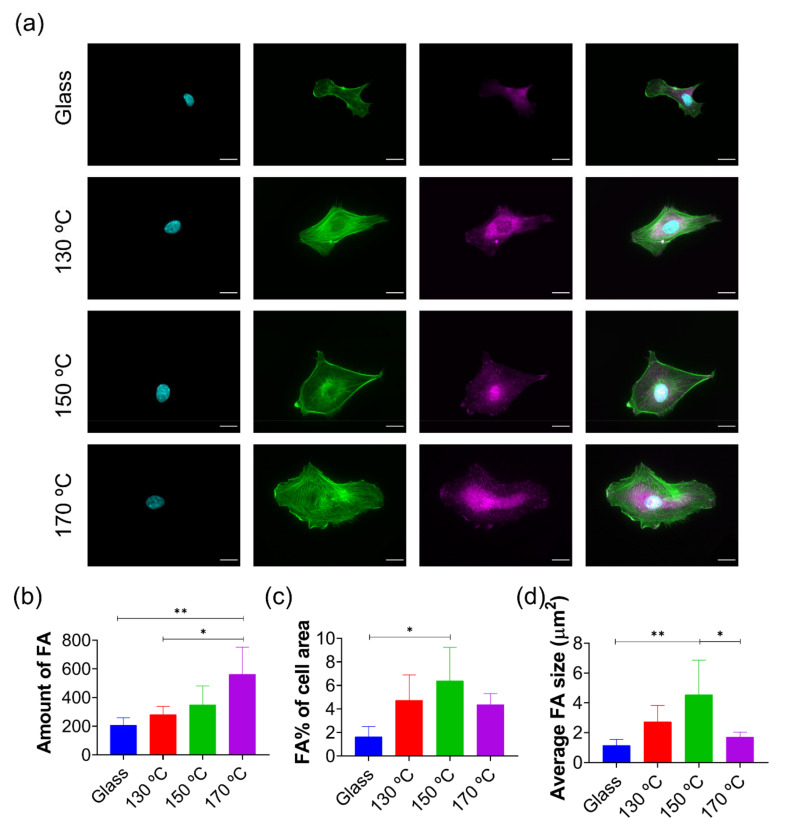
HUVECs FA quantification on PGS substrates. (**a**) Immunofluorescence images of HUVEC nuclei (cyan), actin cytoskeleton (green) and vinculin (magenta) as a focal adhesion (FA) marker of cells cultured after 3 h on glass and different PGS cured materials. Quantification of (**b**) the amount, (**c**) area and (**d**) average size of FA. FA area was calculated as the percentage of the total cell area. All immunofluorescence images share the same scale bar (20 µm). Sample data distributions were analysed through a one-way ANOVA and Bonferroni post hoc multiple mean comparison test, with a *p*-value < 0.05. *, *p* < 0.05 and ** *p* < 0.01.

Subsequently, to investigate the role of different PGS’s curing temperatures in HUVECs early adhesion and FA formation, immunofluorescence quantification was performed. From Figure 3a it can be stated that HUVECs FA expression is mostly revealed in the cytoskeleton periphery and nucleus. The number of FAs increases gradually with curing temperature (Figure 3b). A temperature-dependent trend is also observed in the FA percentage of cell area (cytoskeleton surface) (c) and (d) the relative area of FA compared to the total cell area, using actin quantification. However, 150 °C is the temperature yielding the most remarkable results in this respect, PGS 170 °C performing as PGS 130 °C in terms of FA area, and worse than it is for FA size.

#### 3.2.2. PGS Substrates Protein Adsorption

**Figure 4:**
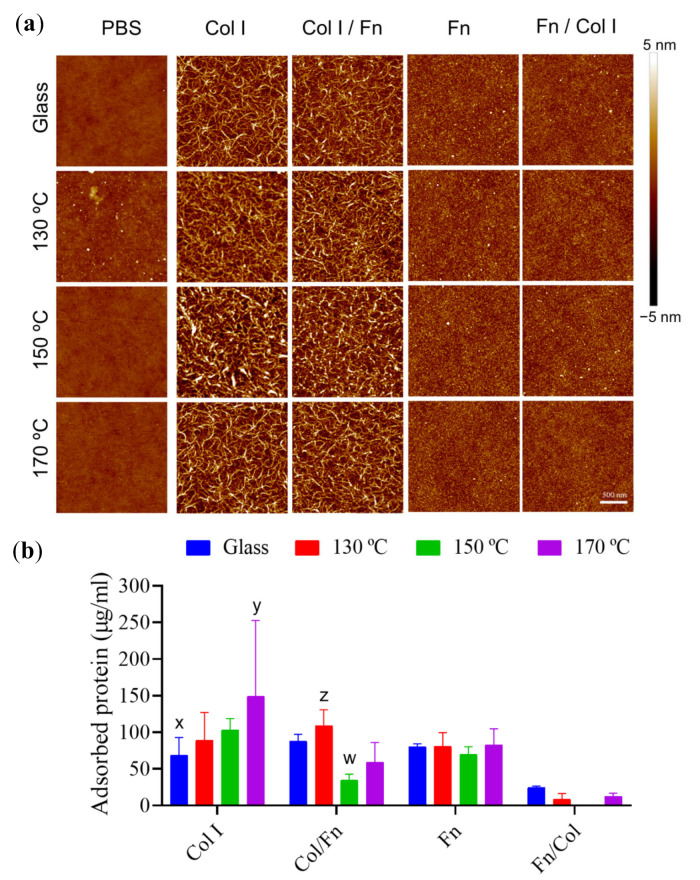
Sequential protein adsorption on PGS substrates. (**a**) Atomic force microscope (AFM) height images of 2 × 2 µm^2^ and (**b**) protein adsorption quantification from microBCA colorimetric technique with single protein (Fn 100 μg/mL or Col I 400 μg/mL) and sequential adsorption assays of Fn and Col I (Fn 100 μg/mL+ Col I 80 μg/mL), and vice versa (Col I 400 μg/mL + Fn 20 μg/mL) on PGS cured at different temperatures. The first column in (**a**) shows the presence of PGS on the glass with no major difference in height for any of the applied curing temperatures. All images share the 500 nm scale bar. Glass covers without spin-coated PGS are shown in (**b**) as control. Sample data distributions were analysed through two-way ANOVA and a Bonferroni post hoc multiple mean comparison test, with a *p*-value of 0.01.

Figure 4a shows the AFM images obtained after sequential and monoprotein adsorption experiments (with PBS as a protein-free control) to determine the effect of curing temperature on the acellular performance of the substrates. Protein adsorption was quantified using the colorimetric test microBCA (Figure 4b). 

AFM results from PBS (Figure 4a) confirm the homogeneity of PGS substrates for all the curing temperatures (with roughness values ranging from 0.2 to 0.3 nm), which sets out the spin-coating technique as a good option for synthetising nanometric coatings of PGS. Col I (Figure 4a, Col I column) appears as a homogeneous and continuous fibrillar coating, with interconnected fibres having a diameter of 20 ± 5 nm in all substrates. This behaviour is particularly outstanding in PGS samples cured at high temperatures (Figure 4b).

There is no significant difference derived from Fn adsorption, as shown in the AFM images, where protein conformation remains globular across all the PGS surfaces tested (Figure 4a Fn column). Protein quantification does not reveal major differences in Fn adsorption, where similar Fn protein outputs were obtained for all PGS samples and glass coverslips.

AFM images from sequential experiments highlighted the non-interchangeable role of the order of protein deposition. On the one hand, when Fn is the first incubated protein, Col I is not able to form its typical fibrillar structures that are formed when Col I is adsorbed in a single step. This is because the Col I-Col I interactions shift to Col I-Fn ones, with Fn already adsorbed onto the surface, as found in [27] where it was studied how Fn binds to Col I via its collagen-binding domain. Since Col I protein concentrations are lesser in the Fn/Col I sequential condition, together with its interaction with Fn, an impairment of its Col I-Col I fibrillar network appears subsequently. This behaviour is alike no matter the temperature set to cure the PGS substrates (Figure 4a, column Fn/Col I). In line with the above, Figure 4b shows that proteins adsorbed in the Fn/Col I experiment are negligible when compared with the rest of the protein coating scenarios.

On the other hand, when Col I is the first protein layer which is adsorbed on PGS substrates, the Col I fibrillar network is formed and remains stable after Fn adsorption (Figure 4a, Col I/Fn column). The amount of protein adsorbed is similar to the values obtained when Col I is used as a single protein coating in all substrates. In the case of higher temperatures (150 °C and 170 °C), denser PGS networks appear to influence the Fn adhesion, which seems to partially desorb the Col I adsorbed in the first stage. This is again the Fn-Col I affinity effect described before, which induces a partial desorption of Col I, by forming soluble Fn-Col I structures in substitution of PGS-Col I. However, Col I cannot be adsorbed in the empty spaces left between Fn proteins, leading to a mix between single protein adsorption and a layer-by-layer coating.

**Figure 5:**
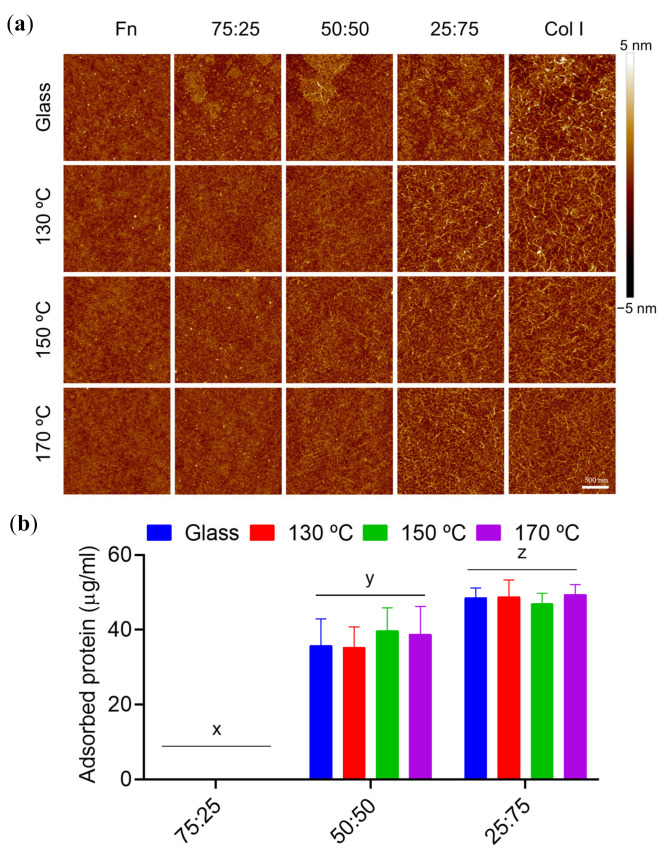
Protein competition adsorption on PGS substrates. (**a**) AFM height images of 2 × 2 µm^2^ and (**b**) protein adsorption quantification from microBCA colorimetric experiments after competition adsorption assays with Fn and Col I on glass and PGS cured at different temperatures. The indicated percentages of intermediate protein mixtures are volumetric ratios of Fn 10 µg/mL over Col I 40 µg/mL. All images share the scale bar of 500 nm. Sample data distributions were analysed through a two-way ANOVA and Bonferroni post hoc multiple mean comparison test, with a *p*-value of 0.0001.

Multiple volumetric proportions of Col I and Fn were then incubated onto PGS substrates and glass to study the effect of protein competition. The chosen concentrations were Col I at 40 μg/mL and Fn at 10 μg/mL at 75:25, 50:50 and 25:75 of Col I:Fn with concentrations of 32.5 μg/mL, 25 μg/mL and 17.5 μg/mL, respectively. These concentrations were selected to meet the requirements of equal concentration of binding sites and similar surface coverage efficiency, according to the recommendations of each protein supplier. AFM images after adsorption assays are represented in Figure 5a together with their corresponding protein quantification in Figure 5b.

Protein quantification results present values of protein adsorption around 50 μg/mL and 30 μg/mL when 25:75 and 50:50 dilutions were respectively incubated onto the PGS substrates. No significant differences were detected between substrates (Figure 5b).

If 75:25 protein dilution was incubated onto the substrates, the amount of protein adsorbed was not high enough to be measured by the colorimetric technique used. These results confirm that 75:25 dilution, with 40 µg/mL for Col I and 10 µg/mL for Fn, leads to Fn-Col I interaction instead of Col I-Col I, avoiding its adsorption throughout the surface of each material.

#### 3.2.3. PGS Substrate-Protein-HUVECs Interaction

**Figure 6:**
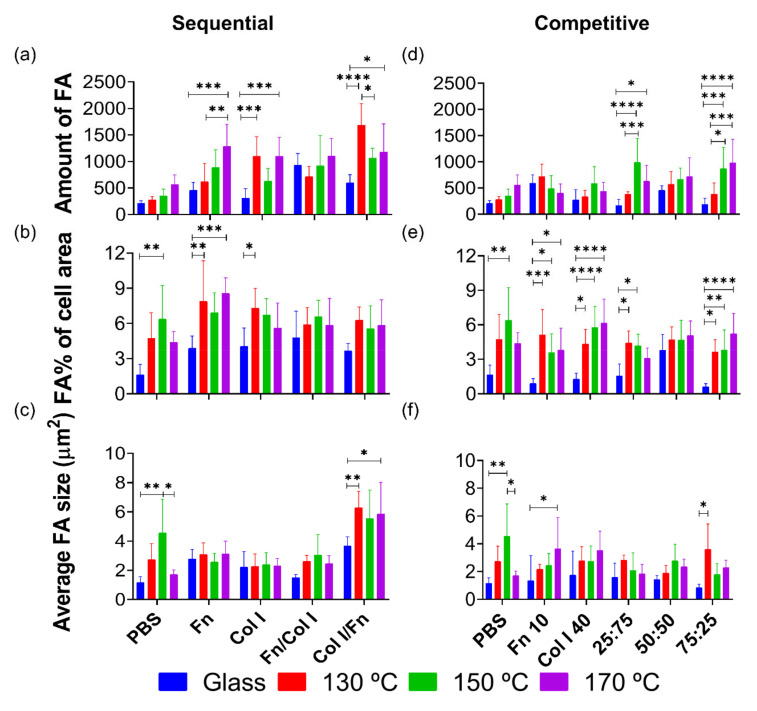
FA parameters of HUVECs cultured on PGS substrates pre-treated in (**a**–**c**) sequential and (**d**‒**f**) competitive protein adsorption experiments. HUVECs fluorescence quantification of (**a**,**d**) number of FA, (**b**,**e**) relative surface of FA compared to the total cell area and (**c**,**f**) average size of FA, from protein sequential and competitive adsorption assays on glass and PGS cured at different temperatures. FA area was calculated as a percentage (%) of the total cell area. Sample data distributions were analysed through a two-way ANOVA and Bonferroni post hoc multiple mean comparison test, with a *p*-value < 0.05. *, *p* < 0.05; **, *p* < 0.01; ***, *p* < 0.001; and ****, *p* < 0.0001.

To investigate the role of PGS-proteins-HUVECs early adhesion and the material’s chemistry effect on FA formation, immunofluorescence quantification was studied, as exposed in Figure 6. All PGS conditions lead to better FA results if compared with glass. Particularly, when Fn is incubated onto the PGS samples, FA related parameters increase with PGS curing temperature since Fn enhances integrins receptors directly related with FA [35]. Thus, the results obtained under Fn/Col I protein incubation can be justified by the Fn-Col I binding effect, which avoids Col I-Col I linkage and blocks its fibrillar structure formation.

In consonance with Col I quantification (Figure 4), showing that more Col I is adsorbed when the curing temperature increases, the amount of FA and the FA% of cell area (Figure 6a,b) becomes higher than the AF observed in glass substrates. This is because of the synergic effect of PGS substrates and the Col I adsorption onto the surface, which produces better cell-material interactions.

Furthermore, Col I/Fn produces a join effect, particularly in the 130 °C condition, where cell response increases its FA. When CoI I is incubated before Fn, Fn is able to fit the free spaces between Col I-Col I fibres by bonding the PGS substrate and generating Col I-Fn interactions, which may increase the amount of binding sites for cell adhesion.

The influence of PGS substrates cured under different temperatures in the competitive incubation of Fn and Col I (Fn 10 μg/mL and Col I 40 μg/mL) can be determined using immunofluorescence FA quantification (Figure 6d–f). Each PGS substrate coated with Fn:Col I mixtures presents significant and better FA outcomes when compared with glass. In this experiment, there is an increasing trend between the amount of FA and its relative cell surface and the curing temperature. Particularly, curing at 150 °C leads to a greater amount of FA when using the 75:25 mixture, although no protein could be detected by the colorimetric techniques used (Figure 5). Nonetheless, AFM images confirm that proteins from the 75:25 dilution are present on the polymer surfaces. In this case, the increase of the amount of FA is produced because of the low-density protein coating, which allows cells to interact with the PGS substrate, promoting cell adhesion as well. Furthermore, the 25:75 mixture, with more content of Col I, leads to an increasingly significant trend of FA number, directly related to the increase of PGS curing temperature. The protein quantification values, together with AFM images from Figure 5, show the same amount of protein adsorbed. All this together indicates that a significant FA increment is produced since HUVECs interact, not only with the proteins adsorbed, but with the PGS substrate itself, therefore producing a synergic effect with that of the curing temperature of the biomaterial.

## 4. Conclusions

A novel PGS synthesis procedure was developed to obtain smooth polymer coatings at a nanometric scale. These structures enable the study of the influence of the chemistry of the PGS networks obtained, with no distorting interactions involving structural variables. PGS coatings, obtained from their pre-polymer, were then cured at different temperatures (130 °C, 150 °C and 170 °C) and were first chemically and biologically characterised. The FTIR spectra show a direct correlation between PGS curing temperature and its reaction kinetics. High temperatures facilitate polycondensations, yielding denser networks with relatively fewer terminal groups. As a result, the wettability of the substrates, quantified by their WCA, decreases slightly. Accordingly, the surface tension increases, the substrates cured at 150 °C showing the maximum value on account of their higher dispersive component.

AFM images, together with microBCA results, reveal protein-material interactions and the amount of protein adsorbed by each surface. The adsorption of a single protein such as Fn is not dissimilar on the set of substrates, always displaying a cluster distribution. In the case of Col I, the chemical variations of the obtained networks are expressed: higher curing temperatures increase its adsorption, in turn promoting Col I-Col I interactions that generate a homogeneous fibrillar Col I network coating. However, a sequential adsorption of these proteins revealed that Col I does not adsorb if Fn is present on the surface before, arguably due to Fn-Col I interactions that yield soluble complexes. Conversely, when substrates are first coated with Col I, and particularly those cured at 130 °C, Fn can interact with Col I thanks to its smaller size, fitting through the empty spaces within the Col I network that had formed previously. This scenario yields heterogeneous non-conclusive results with respect to the influence of the curing temperature on a sequential protein adsorption. Adsorption of proteins from Fn:Col I mixtures (competition assays) at different volumetric proportions does not yield significant differences between samples. As the quantity of Col I increases at the expense of Fn, the total amount of protein adsorbed increases. This is due to the major PGS substrates-Col I affinity and the inhibitory effect of Fn in the presence of Col I.

With regard to in vitro cultures of HUVECs on PGS substrates, absence of cytotoxicity was confirmed after 1, 3, 5 and 7 days using the colorimetric AlamarBlue^TM^ test. Immunofluorescence results reveal differences between PGS materials regarding the presence of the total amount of FA, quantified via vinculin fluorescence expression. When the PGS curing temperature is increased, the number of HUVECs FAs improves alongside due to the interaction of the PGS networks, with greater values of surface tension, and the aqueous media fraction of protein dilutions during its incubation. When proteins were adsorbed prior to cell cultures, all PGS networks showed a better cell response when compared with glass. Particularly, the Col I/Fn combination has a synergic effect, which increased cell FA values, particularly on samples cured at 130 °C. When mixtures of these proteins were incubated in one single step, specifically the 25:75 Fn:Col I mixture, rich in Col I, a significant increasing trend was found for the FA number, directly related to the increase of the curing temperature.

All in all, it can be stated that Col I has a stronger interaction with PGS substrates than Fn, and its adsorption is promoted by less polar, denser PGS networks cured at high temperatures. An adsorption with this protein, prior to a cell culture, improves the parameters related to cell adhesion, but it seems to be of special interest on samples cured at 130 °C.

## Figures and Tables

**Table 1:** Surface energy components and water contact angles (WCAs) of glass and PGS samples cured at different temperatures, calculated from contact angle data, and using the OWRK equation.

Material	*σ_d_* (mN/m)	*σ_p_* (mN/m)	*σ_total_* (mN/m)	WCA (°)	*σ_p_/σ_total_*
Glass	16.43	34.03	50.47	45.30 ± 4.82	0.67
130 °C	14.48	18.48	32.96	71.16 ± 1.43	0.56
150 °C	17.72	18.08	35.8	68.07 ± 3.94	0.51
170 °C	15.5	19.84	35.34	67.46 ± 3.94	0.56

## Data Availability

The data presented in this study are available on request from the corresponding author.
